# Beyond the Storm: CXCL9 and the New Era of Precision Hemophagocytic Lymphohistiocytosis Diagnostics

**DOI:** 10.3390/diagnostics16172783

**Published:** 2026-08-30

**Authors:** Thomas F. Fusillo, Johnson M. Liu

**Affiliations:** 1 Department of Medicine, Icahn School of Medicine at Mount Sinai, Mount Sinai Morningside/West, New York, NY 10019, USA; fusillot@mskcc.org; 2 Department of Medicine, Memorial Sloan Kettering Cancer Center, New York, NY 10065, USA; 3 Department of Medicine, Division of Hematology and Medical Oncology, Icahn School of Medicine at Mount Sinai, New York, NY 10029, USA

**Keywords:** hemophagocytic lymphohistiocytosis (HLH), CXCL9, interferon-gamma, macrophage activation syndrome (MAS), cytokine storm, biomarker, inflammation

## Abstract

Hemophagocytic lymphohistiocytosis (HLH) is associated with high mortality, underscoring the importance of rapid diagnosis and treatment. However, accurate and timely diagnosis is challenging due to its similarities with other acute inflammatory conditions. Traditional diagnostic frameworks rely on fever, lymphadenopathy, cytopenias, hyperferritinemia, hypofibrinogenemia, and related laboratory abnormalities, many of which may reflect either HLH or the underlying condition that triggered or mimics it. Newer diagnostics have emerged that are based on the underlying disease biology, particularly interferon-gamma (IFN-γ)-driven immune activation. C-X-C motif chemokine ligand 9 (CXCL9), a downstream marker of IFN-γ activity, has emerged as a promising adjunctive biomarker for HLH diagnosis, risk stratification, monitoring treatment response, and detection of disease reactivation. This narrative review examines the current evidence surrounding CXCL9 and its role in HLH.

## 1. Introduction

Hemophagocytic lymphohistiocytosis (HLH) is a syndrome characterized by inappropriate immune activation and inflammation. Its name derives from the characteristic histopathologic finding of macrophages phagocytosing hematopoietic cells within the reticuloendothelial system. HLH is classically split into primary and secondary forms: primary HLH is a congenital disorder that usually presents in childhood, whereas secondary HLH is typically triggered by underlying medical conditions later in life.

Secondary HLH was the first form widely described in a case series published in 1939. The authors called the syndrome histiocytic medullary reticulosis and described it as “fever, wasting and generalized lymphadenopathy […] associated with splenic and hepatic enlargement and in the final stages jaundice, purpura and anemia with profound leukopenia” [1]. Primary HLH was later widely described in a case series, published in 1952, of three infants [2].

As recognition of HLH has increased, its underlying etiologies have been more precisely defined. Primary, or familial, HLH is driven by genetically determined defects in cytotoxic natural killer (NK) and T-cell function through mutations in genes including *PRF1*, *UNC13D*, *STX11*, and *STXBP2* [3,4]. Secondary HLH remains a commonly used term, but cases are increasingly classified by the underlying trigger, most commonly infection, malignancy (particularly lymphomas and leukemias), autoimmune (referred to as macrophage activation syndrome or MAS), chimeric antigen receptor (CAR) T-cell therapy, and medications [5].

Mortality varies substantially by underlying cause—ranging from under 25% in infection-associated HLH to over 70% in malignancy-associated or untreated primary HLH—but all forms are associated with significant mortality, necessitating urgent diagnosis and treatment [5,6,7,8,9]. Nevertheless, HLH remains a diagnostic challenge due to overlapping presentations and biomarkers with other inflammation-driven diseases. The two predominant diagnostic frameworks are the HLH-2004 criteria and the HScore, both of which utilize a variety of clinical and laboratory measures, many of which are readily available (Table 1) [10,11]. Although these systems are easy to use, many of their components lack specificity for HLH; thus, they can still yield false positives.

C-X-C motif chemokine ligand 9 (CXCL9) is an interferon-gamma (IFN-γ)-induced chemokine that has emerged as a promising biomarker with increased specificity for the diagnosis of HLH. This narrative review examines the current evidence for CXCL9 in HLH diagnosis, differentiation from mimicking conditions, prognostication, treatment monitoring, and detection of disease reactivation.

## 2. Pathophysiology of the IFN-γ–CXCL9 Axis in HLH

**Figure 1:**
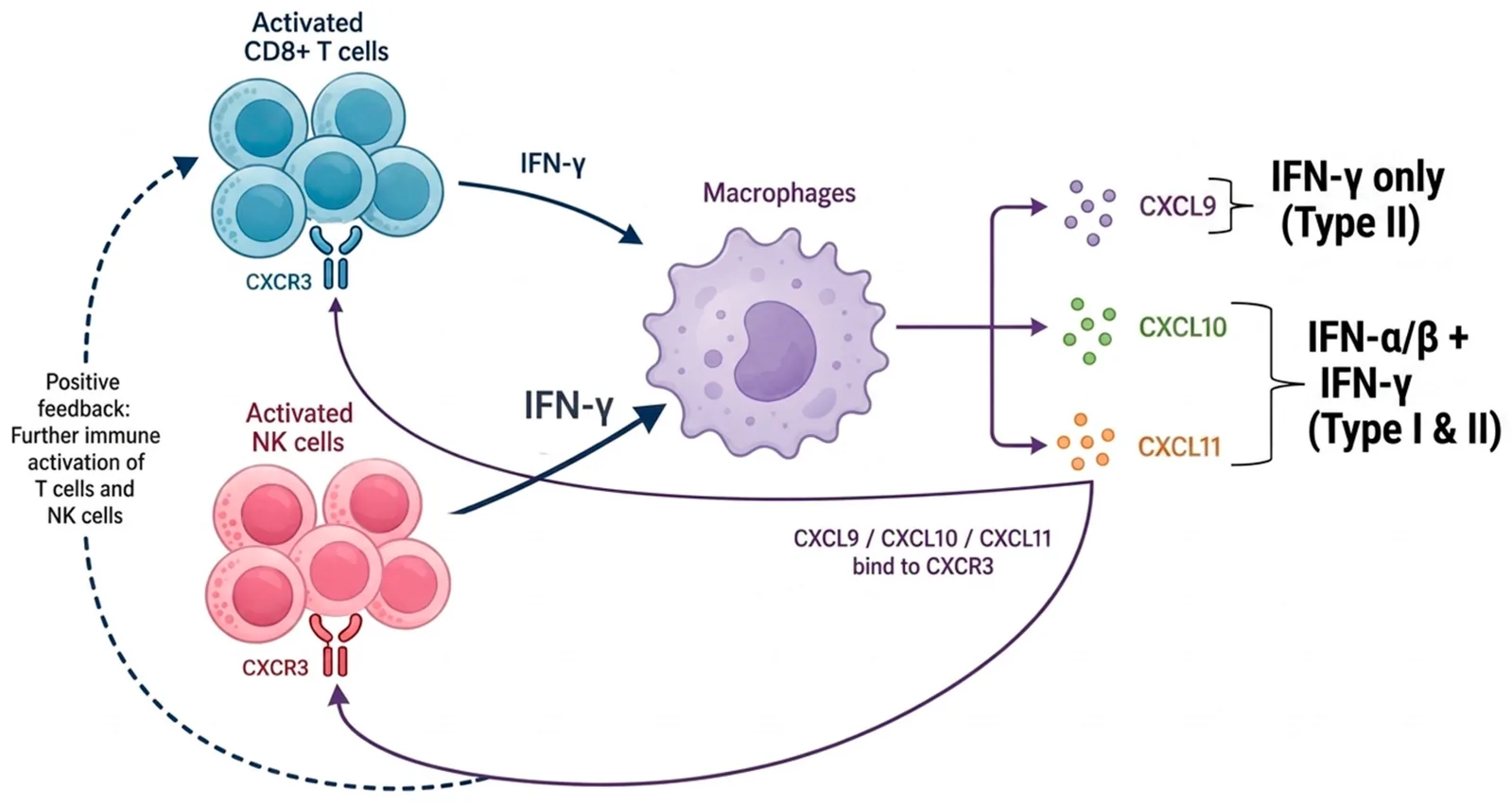
Interferon-gamma–CXCL9 axis in HLH.

The pathophysiology of HLH is driven primarily by impaired lymphocyte cytotoxicity, preventing NK and CD8+ T cells from eliminating infected or malignant target cells [7]. This in turn leads to persistently activated lymphocytes, which release cytokines including tumor necrosis factor-alpha, interleukin-1β, interleukin-6, interleukin-10, interleukin-18, and interferon-γ [5,7,13,14]. IFN-γ activates macrophages, which further stimulate lymphocyte activation and cytokine release, creating a positive feedback loop (Figure 1). This sustained persistent cytokine release and immune activation produces the hyperinflammatory state in HLH that eventually leads to tissue injury and organ failure [4,5,7].

In response to NK and T-cell production of IFN-γ, myeloid cells—predominantly macrophages—produce CXCL9, CXCL10, and CXCL11, all of which are ligands of the C-X-C motif chemokine receptor 3 (CXCR3) found on NK and T cells [15]. IFN-γ, a type II interferon, is distinct from type I interferons such as IFN-α and IFN-β which tend to be produced earlier in the innate immune response, particularly in response to viral infections [16]. This is an important distinction because CXCL9 is only induced by type II interferons (IFN-γ), while CXCL10 and CXCL11 are induced by both type I (IFN-α/β) and type II interferons (IFN-γ) [17]. Therefore, CXCL9 is the most specific downstream marker of IFN-γ activity compared with CXCL10 and CXCL11.

Direct measurement of IFN-γ levels has several limitations. First, serum IFN-γ may not always be elevated in patients with HLH. In a study of 53 patients with HLH, Luo et al. found that 17% exhibited normal IFN-γ levels, whereas 98% had elevated CXCL9 levels [18]. This suggests that IFN-γ is a less sensitive marker for HLH. Second, there may be a large discrepancy in tissue versus serum IFN-γ levels. Prencipe et al. demonstrated in three patients with HLH who received liver biopsies that IFN-γ pathway gene expression was elevated in the tissue samples but IFN-γ levels were undetectable in the serum of two out of three patients [19]. Furthermore, IFN-γ levels may decrease after treatment faster than CXCL9 levels, potentially limiting its usefulness for response monitoring.

In the Luo et al. study, 7 days after treatment, 85% of patients had normal levels of IFN-γ versus 35% with normal levels of CXCL9 [18]. Thus, CXCL9 may be more useful in monitoring treatment response and detecting early progression or reactivation.

Importantly, CXCL9 appears to function as a biomarker rather than a driver of HLH. Diamond et al. induced HLH and MAS in CXCL9-knockout mice (cxcl9^−^/^−^) and in mice with CXCR3 inhibition. In both groups, there was no improvement in disease parameters compared to normal controls [20]. These findings suggest that the CXCL9–CXCR3 interaction itself is not a major pathogenic driver of HLH or MAS. Instead, elevated CXCL9 reflects increased IFN-γ signaling, supporting its role as a reliable surrogate marker of IFN-γ pathway activity.

## 3. CXCL9 in the Diagnosis of HLH

The laboratory markers included in the HLH-2004 and HScore criteria are hemoglobin, platelets, white blood cells, triglycerides, fibrinogen, NK cell activity, ferritin, soluble interleukin-2 receptor (sCD25), and aspartate aminotransferase (AST) [10,11]. Although many of these markers are sensitive, they often lack specificity, which makes HLH difficult to distinguish from severe infection, malignancy, rheumatologic disease, and other inflammatory syndromes. CXCL9 may help improve the diagnostic specificity by reflecting IFN-γ signaling rather than non-specific inflammation. Ferritin, for example, may be elevated in iron overload, malignancy, sepsis, and other inflammatory states. Furthermore, the 2022 European Alliance of Associations for Rheumatology (EULAR) and American College of Rheumatology (ACR) HLH guidelines specifically acknowledge that the existing diagnostic criteria can be confounded by conditions such as malignancy and sepsis [21].

Because CXCL9 is a surrogate of IFN-γ activity, it can be elevated across the spectrum of IFN-γ-driven pathology rather than HLH alone. Increased CXCL9 has been described in MAS, systemic juvenile idiopathic arthritis, adult-onset Still’s disease, sarcoidosis, active tuberculosis, lymphomas, HIV, Epstein–Barr virus (EBV), and sepsis [22,23,24,25,26,27,28,29,30]. Given this near-universal positivity for these IFN-γ-driven conditions, the magnitude of CXCL9 elevation—rather than mere positivity—may carry the discriminatory signal, as reflected in the markedly higher levels seen in HLH than its mimics. Additionally, no study has included all of these conditions in the same cohort; thus, cross-condition CXCL9 comparisons are indirect.

### 3.1. Diagnostic Performance Characteristics

Similar to the laboratory criteria included in HLH-2004 and the HScore, CXCL9 appears to be highly sensitive for the diagnosis of HLH. In one study, 98% of patients with HLH of various subtypes had an elevated CXCL9 [18]. However, its specificity is more context-dependent. CXCL9 is a marker of increased IFN-γ pathway activation; thus, it may be elevated in any IFN-γ-driven inflammatory state. For example, in a cohort of 143 patients (15 adult and 128 pediatric) with cytokine storm syndromes, CXCL9 was significantly elevated in primary (familial) HLH, virus-associated HLH, lymphoma-associated HLH, and Kawasaki disease-associated MAS, but only moderately elevated in other etiologies such as multisystem inflammatory syndrome in children and toxic shock syndrome [31].

The magnitude of CXCL9 elevation may therefore be more informative than elevation alone. In one of the largest multicenter adult HLH cohorts, Rocco et al. studied 126 HLH-positive and 45 HLH-negative hospitalized patients undergoing evaluation for suspected HLH. Median CXCL9 was 1678 pg/mL (interquartile range [IQR] 636–4274 pg/mL) in HLH-negative patients compared with 16,102 pg/mL (IQR 5664–90,749 pg/mL) in HLH-positive patients (*p* < 0.001), highlighting the difference in CXCL9 levels in HLH compared to mimicking conditions [32]. This means that in this study, patients diagnosed with HLH had CXCL9 levels 10 times those in patients who presented with inflammation-like diseases but were ultimately HLH-negative. Median CXCL9 did not differ significantly between malignancy-associated HLH (16,106 pg/mL) and non-malignancy-associated HLH (16,098 pg/mL), suggesting that marked CXCL9 elevation may occur across HLH subtypes [32].

However, CXCL9 does not appear to outperform other biomarkers in every diagnostic context. In a study of 120 patients, including 14 with confirmed HLH, ferritin, interleukin-18, and glycosylated ferritin had the highest areas under the curve (AUCs) for discriminating HLH across subtypes [33]. Notably, among confirmed HLH cases, CXCL9 was significantly higher in malignancy-associated HLH than in infection-associated HLH, although this comparison included only four malignancy-associated cases [33].

An emerging application of CXCL9 is in the diagnosis of central nervous system (CNS) involvement in HLH (CNS-HLH). CNS-HLH occurs in approximately 40% of patients with HLH and often mimics other neuroinflammatory disorders such as encephalomyelitis and multiple sclerosis [34,35]. Chandra et al. studied proteomics in the cerebrospinal fluid (CSF) of patients with CNS-HLH and other neuroinflammatory disorders (ONID) and observed elevated CXCL9 levels in 85% of CNS-HLH samples versus 5% in ONID samples and a median CXCL9 level of 223.7 ng/mL in CNS-HLH versus 15.5 ng/mL in ONID (*p* ≤ 0.001) [35]. In a separate pediatric cohort, Zhao et al. also observed significantly higher CNS CXCL9 levels among patients with CNS-HLH compared to HLH without CNS involvement and a cutoff value of 19.54 pg/mL with an AUC > 0.9 and sensitivity and specificity > 80% [36]. These findings extend the diagnostic utility of CXCL9 beyond serum to the CSF, although larger cohorts are needed.

### 3.2. CXCL9 Versus IFN-γ as a Diagnostic Marker

As previously mentioned, CXCL9 levels provide more useful information than IFN-γ in many scenarios surrounding diagnosis of HLH. From a technical perspective, this may be due to IFN-γ being present primarily in tissue, while CXCL9 is more reliably detected in the serum [37]. However, one situation that appears to favor IFN-γ is in discerning between EBV infectious mononucleosis and EBV infection-associated HLH (EBV-HLH). Luo et al. observed an AUC of 0.926 for IFN-γ versus 0.568 for CXCL9 (*p* < 0.001) in this context [18].

#### EBV-HLH Versus Systemic EBV-Positive T-Cell Lymphoma

A particularly challenging diagnostic scenario is in distinguishing systemic EBV-positive T-cell lymphoma of childhood (SEBVTCL) from EBV-HLH. The World Health Organization (WHO) classifies SEBVTCL within a spectrum of EBV-positive NK/T-cell lymphoid proliferations and lymphomas along with systemic chronic active EBV disease (CAEBV) but does not explicitly mention HLH or EBV-HLH [38]. These entities frequently present with overlapping clinical presentations including fever, hepatosplenomegaly, cytopenias, coagulopathy, and elevated EBV viral loads, making a timely accurate diagnosis difficult [39,40]. While clonal T-cell receptor (TCR) gene rearrangement is a hallmark of T-cell malignancies, including SEBVTCL, these rearrangements are often also observed in EBV-HLH, further blurring the line between these entities [41]. Additionally, SEBVTCL frequently presents with HLH [39,42]. As mentioned, Luo et al. looked at CXCL9 versus IFN-γ when diagnosing EBV infectious mononucleosis versus EBV-HLH, but there is no published data on the utility of CXCL9 when distinguishing EBV-HLH from SEBVTCL [18]. Whether the magnitude of CXCL9 elevation can help distinguish EBV-HLH from SEBVTCL warrants investigation.

### 3.3. Diagnostic Cutoff Values

There is little data supporting a single CXCL9 diagnostic cutoff value for HLH, and no society guidelines support a numeric value [21,43]. However, the North American Consortium for Histiocytosis (NACHO) HLH guidelines note that elevated CXCL9 “should be seen in untreated cases of HLH” [43]. Luo et al. reported an AUC of 0.948 for elevated CXCL9 in distinguishing HLH from non-EBV infection, although no specific cutoff was proposed [18]. In a small study of 15 patients with lymphoma-associated HLH (nine B-cell and six T/NK-cell) compared with 18 patients with lymphoma (16 B-cell and two T/NK-cell) but without HLH, CXCL9 > 5000 pg/mL had 100% sensitivity and 95% specificity for diagnosis of HLH [44]. Rocco et al. proposed CXCL9 > 16,100 pg/mL for prognostication but did not propose a diagnostic cutoff (Table 2) [32]. Notably, the recently updated HLH-2024 diagnostic guidelines for familial HLH do not include CXCL9 among its criteria (Table 1), nor does the HScore [10,12]. This omission likely reflects the current absence of prospectively validated thresholds. Overall, these data suggest that CXCL9 is a highly sensitive marker that adds diagnostic value through its specificity for IFN-γ driven pathology, but further validation of cutoff values remains an important unmet need.

### 3.4. CXCL9 as an Adjunct to Existing Biomarkers and Scoring Systems

No single biomarker is both highly sensitive and specific for HLH and neither HLH-2004, HLH-2024, nor the HScore incorporates CXCL9. The pathophysiology of HLH is driven by the activation of multiple biological pathways, including NK/T-cell, macrophage, inflammasome, and the IFN-γ pathway [7,47]. Therefore, a multimodal diagnostic model incorporating markers representative of the several pathophysiological pathways activated, including CXCL9 (IFN-γ), sCD25 (T-cell), IL-18 (inflammasome), and ferritin/glycosylated ferritin (macrophage), may improve diagnostic specificity while preserving sensitivity (Table 3). The 2022 EULAR/ACR Points to Consider acknowledge these markers, in addition to other cytokines, as having individual utility in diagnosing HLH, but do not propose any combined model [21].

Debaugnies et al. showed that combining a laboratory marker with a pre-existing scoring system can improve specificity. The authors did this by adding IL-18 to the HScore, yielding a specificity of 96% compared to 89% with the HScore alone and maintaining a 93% sensitivity [33]. A similar framework adding CXCL9 may also yield additional benefit and warrants investigation. This approach of adding cytokines representative of the pathways underlying HLH represents a promising opportunity to further refine scoring systems.

## 4. CXCL9 as a Prognostic Marker

After HLH is diagnosed, prompt treatment is essential. Mortality estimates vary by age and subtype, but overall mortality is approximately 30–50% [5,48,49]. Because treatment ranges from corticosteroids to etoposide-based chemotherapy to targeted IFN-γ blockade, early identification of patients at highest risk of mortality could help guide treatment intensity and monitoring.

### 4.1. CXCL9 and Mortality Prediction

The largest multicenter study on CXCL9 for HLH prognostication was published by Rocco et al. in 2026. The study included 126 patients meeting HLH criteria across various subtypes. Using unbiased decision-tree modeling, the authors identified CXCL9 > 16,100 pg/mL as the single optimal predictor of inpatient mortality (Table 2). This cutoff was also significantly associated with 90-day mortality [32]. In multivariable analysis adjusting for CXCL9, age, sex, race/ethnicity, malignancy, and treatment, patients with CXCL9 > 16,100 pg/mL had a significantly higher 90-day mortality, with a hazard ratio (HR) of 3.0 (95% confidence interval [CI] 1.6–5.6) with most deaths occurring within the first 30 days [32].

Different HLH subtypes have differing pathophysiology, which could impact the prognostic capability of CXCL9. In the same study, Rocco et al. then split the cohort into malignancy-associated HLH and non-malignancy-associated HLH sub-cohorts and once again observed significantly increased 90-day mortality rates for patients in each group with CXCL9 > 16,100 pg/mL [32].

Finally, Rocco et al. looked at CXCL9 as a continuous variable to determine if a rising CXCL9 was associated with increased mortality. The authors found that for every 5000 pg/mL increase in CXCL9, 90-day mortality odds increased by 4% (*p* = 0.001). Given the wide range of CXCL9 levels in the HLH-positive patients in this study (IQR 5664–90,749 pg/mL), this could imply large variation in mortality risk among patients with HLH based on CXCL9 [32]. Altogether, these findings suggest that CXCL9 could be used at the time of HLH diagnosis to identify high-risk patients while also providing a rationale for trending CXCL9 during the early phases of the disease.

### 4.2. CXCL9 in Monitoring Treatment Response

Emapalumab is a fully human IgG1 anti-IFN-γ monoclonal antibody that is used to treat primary/familial HLH and HLH/MAS associated with Still’s disease. In the phase 2/3 trial of emapalumab in children with primary HLH, CXCL9 levels were trended after administration of emapalumab and demonstrated a 30% decrease from baseline levels by day 5 post-treatment. Logistic regression also demonstrated that lower CXCL9 levels were associated with an increased probability of response at end of treatment (*p* = 0.03) [50].

In the REAL-HLH study, a retrospective cohort study of 17 patients treated with emapalumab for malignancy-associated (secondary) HLH, 75% (six out of eight patients) had abnormal CXCL9 levels prior to therapy. At a median of 21 days (IQR 9–68 days) after emapalumab treatment, 88% (seven out of eight patients) achieved normal CXCL9 levels. In this study, the 90-day survival probability was 35% (95% CI 15–57%) [51]. However, this study did not have a control/comparison arm; thus, whether these normalizations in CXCL9 levels represented beneficial treatment response or were a direct result of emapalumab is difficult to determine.

A larger study looked at CXCL9 during treatment response of 77 patients with secondary HLH treated with emapalumab (n = 54), ruxolitinib (n = 18), or both (n = 5). The authors used decision-tree analysis to identify that CXCL9 ≥ 3500 pg/mL at treatment initiation was associated with improved overall survival (*p* = 0.03). HScore, ferritin, and sCD25 were not significantly associated with improved overall survival [45]. These findings suggest that higher CXCL9 levels prior to treatment may indicate an IFN-γ-predominant mechanism and therefore a greater likelihood of response to anti-IFN-γ-directed therapy. This represents an important interpretive paradox: high CXCL9 at initial presentation may mark severe, high-risk disease, whereas high CXCL9 at the time of anti-IFN-γ-directed treatment may identify a more targeted inflammatory phenotype. In other words, CXCL9 may function as a severity marker in untreated HLH and as a predictive biomarker when therapy directly interrupts the pathway it reflects. In a similar study of 15 patients (one primary, 14 secondary) treated with ruxolitinib, emapalumab, and dexamethasone (RED), CXCL9 and interleukin-18 were significantly higher prior to therapy in patients who responded to RED compared to those who had disease progression (*p* = 0.026 for CXCL9; *p* = 0.019 for interleukin-18) [46].

The 2022 EULAR/ACR guidelines for the diagnosis and management of HLH/MAS state that CXCL9 should be monitored during disease management, although less frequently than conventional markers such as ferritin and c-reactive protein (CRP). These guidelines do not specify CXCL9 thresholds but note that CXCL9 “may be particularly useful for monitoring treatment response to IFN-γ-blocking therapies” [21]. Nevertheless, more data is needed to strengthen society guidelines surrounding the use of CXCL9 in monitoring treatment response.

### 4.3. CXCL9 in Detecting Disease Reactivation

HLH reactivation is more common in primary HLH but can also occur in secondary HLH [7]. Reactivation is a serious concern in patients with HLH due to its high mortality, although it varies by etiology. These mortality rates are difficult to estimate but one study observed a 5-year mortality of 75% in patients with reactivation of HLH [52]. A special report published in Blood estimated mortality in relapsed or refractory HLH in adults to be 20–88%, specifically mentioning that EBV-associated HLH reactivations can be common but generally respond well to intensified treatment [53].

Luo et al. monitored cytokine levels in 20 of the 53 HLH patients following therapy. Among 13 newly diagnosed patients with secondary HLH, seven experienced reactivation within 1 year. Patients who reactivated had higher median post-treatment CXCL9 levels than those who did not (11,375 vs. 599 pg/mL; *p* = 0.001). CXCL9 remained elevated in all patients who later reactivated and normalized only in patients without reactivation, yielding 100% specificity for predicting sustained remission in this cohort. By contrast, IFN-γ normalized in 71% of patients who still went on to reactivate [18]. These findings suggest that CXCL9 is superior to IFN-γ for identifying potential HLH reactivation and that persistently elevated CXCL9 may be a warning sign for disease recurrence, although the evidence is limited by the study’s small single-center pediatric cohort.

Limited societal guidance exists regarding the role of CXCL9 in monitoring patients during and after treatment, as well as in identifying HLH reactivation. However, ACR/EULAR guidelines on the diagnosis and management of HLH and MAS acknowledge that CXCL9 may be useful in monitoring patients with HLH, particularly in those receiving IFN-γ-blocking therapies [21]. Larger cohort studies are needed to validate CXCL9 thresholds for predicting reactivation across HLH subtypes.

## 5. Practical Considerations and Current Limitations

Although CXCL9 is a promising biomarker for HLH, its clinical utility depends on feasibility in routine practice. Cost, turnaround time, and assay availability may limit implementation at some institutions. While the test has become more widely used over the past several years, it remains costly and time-consuming in many settings [54].

### 5.1. Assay Availability and Standardization

There are multiple laboratory methods for measuring CXCL9, including manual enzyme-linked immunosorbent assay (ELISA), semiautomated microfluidic ELISA, and fully automated immunoassay [55,56,57,58]. The most common of these is the manual ELISA. Due to cost and frequency of use, many institutions send samples to outside laboratories for processing [54]. Fortunately, CXCL9 levels in serum are relatively stable up to 24 h at room temperature, such as during shipping to outside laboratories [59].

A common CXCL9 ELISA utilizes a sandwich enzyme immunoassay technique (such as R&D Systems Quantikine ELISA Kit, Catalog #DCX9000) [55]. This entails the sample being added to a microplate with a monoclonal antibody specific to CXCL9, adding an enzyme-linked polyclonal antibody specific for the CXCL9-antibody complex, adding an activating substrate, and measuring the intensity of light emitted [55]. This protocol takes about 5 h if completed consecutively, and, in some cases, costs the performing laboratory over $700 per test, not including labor [55].

Herskovits et al. at Memorial Sloan Kettering Cancer Center, an institution with high CXCL9 testing volume, demonstrated that by insourcing via semiautomated microfluidic ELISA, time to result was decreased by approximately 2 days and came with an annual cost savings of around $140,000 [54]. A semiautomated microfluidic ELISA, such as Ella Automated Immunoassay System (Catalog #60–100) used by Herskovits et al., yields results in around 90 min, with much less manual labor, and can include additional cytokines [54,57]. Including additional cytokines such as soluble interleukin-2 receptor (sCD25) can help provide more robust information to the clinical team without the added laboratory time delay.

The newest generation of CXCL9 laboratory technology is fully automated assays. Hasegawa et al. developed a fully automated immunoassay that demonstrated a coefficient of variation of 7% and a limit of quantitation of just 2.2 pg/mL [58,60]. Although fully automated platforms may improve reproducibility, standardization remains a significant hurdle.

Several methods exist for quantifying CXCL9 levels, but none are approved for clinical use by the Food and Drug Administration (FDA). This likely reflects the need for improved assay standardization and large-scale validation studies establishing reliable universal reference ranges. Importantly, there are no published data on CXCL9 assay standardization, but there are several studies that look at other cytokine assays that still provide valuable insight [61,62,63]. These studies generally show acceptable reproducibility within the same assay platform but substantial variability across manufacturers. This variability may partly explain the wide range of proposed CXCL9 diagnostic and prognostic cutoffs in the published literature [18,32,33,44,45,46].

### 5.2. Limitations of the Current Evidence

The current evidence base for CXCL9 in HLH is limited by small sample sizes, retrospective study designs, and frequent single-center cohorts. To the best of our knowledge, no prospective validation studies or randomized controlled trials have evaluated CXCL9 as a diagnostic or prognostic marker. Rocco et al. is the largest multicenter study in this area to date and included 126 patients with HLH but was retrospective [32]. Many other studies included substantially fewer patients, limiting statistical power and generalizability [18,33,44,46].

A fundamental challenge of nearly all HLH studies is the limitation of the established diagnostic criteria, including HLH-2004 and the HScore [10,11]. Many of the components of these criteria lack specificity and can apply to a broad range of diagnoses, making accurate and timely diagnosis of HLH difficult [64,65]. Therefore, evaluating a new biomarker such as CXCL9 against imperfect pre-existing diagnostic criteria makes it difficult to determine whether discordant cases represent false positives or negatives of CXCL9 or of the reference standard.

CXCL9 can also be elevated due to other underlying etiologies besides HLH such as graft-versus-host disease (GVHD), hematologic malignancies, and sepsis [66,67,68,69,70]. Most studies to date have compared HLH to a limited set of control conditions, and broader comparisons against the full spectrum of IFN-γ-driven diseases are lacking. This is important in practice because many of the conditions that frequently overlap with or mimic HLH in clinical practice—such as sepsis and GVHD—are also associated with elevated CXCL9 levels.

#### CXCL9 Across Ages

Another limitation is the lack of comparison of CXCL9 performance across age groups. This is particularly important because CXCL9 levels in healthy individuals increase with age [71,72,73,74]. One study observed a particularly high jump in CXCL9 levels in healthy individuals ages 70–74 and then again at ages 85–89 [72]. This age-associated increase may confound proposed diagnostic and prognostic cutoff values, particularly when extrapolating between pediatric and various adult populations.

Interestingly, while CXCL9 levels increase with age in healthy adults, the opposite is true in children. Sepiashvili et al. demonstrated in a healthy cohort of ages 1 to 19 that there was a gradual concentration decrease with age, yielding an upper reference limit of 1215 pg/mL (90% CI 1083–1344 pg/mL) among 1–8 years and 905 pg/mL (90% CI 647–1069 pg/mL) among 9–19 years [75]. Combined with data in adults, this suggests a U-shaped relationship between CXCL9 and age. Thus, a fixed cutoff risks lower specificity in young children and older adults, both groups with higher baseline CXCL9 levels. These findings argue against a single universal cutoff and favor age-based reference intervals.

## 6. Future Directions

Future studies will ideally make CXCL9 a more definitive clinically actionable biomarker. The first step towards this paradigm shift is prospective validation in diverse HLH etiologies including pediatric and adult patients, malignancy-associated HLH, infection-associated HLH, HLH/MAS, EBV-HLH (particularly when distinguishing from SEBVTCL), post-transplant HLH, CAR T-cell-associated hyperinflammation, and patients with HLH mimics such as sepsis and GVHD. These studies should evaluate CXCL9 alongside established diagnostic frameworks and guidelines rather than in isolation, because the utility of CXCL9 is likely additive.

Another priority is standardizing assay methodology and establishing CXCL9 cutoffs. Diverse prospective validated studies will help establish these cutoffs, but standardizing assay methodology also requires a robust technical review and quality control. Cross-platform calibration will also improve assay standardization and make FDA approval more promising. Future research should therefore report assay platform, sample type, timing relative to treatment, and comparator populations to allow for meaningful comparison across studies.

Clinically, CXCL9 should also be studied as part of multimarker and dynamic-response models. Existing markers and models are already useful and therefore incorporating CXCL9 with known HLH biomarkers such as ferritin, sCD25, fibrinogen, interleukin-18, and cytopenias will likely provide more valuable insight and increase sensitivity and specificity of these models. Given the existing data on the utility of trending CXCL9 during disease treatment and monitoring for recurrence, protocols that define what change in value should prompt escalation or de-escalation of therapy would be particularly valuable.

An additional area requiring investigation is the role of CXCL9 in immune effector cell-associated HLH-like syndrome (IEC-HS), an increasingly recognized complication of CAR T-cell therapy [76]. IEC-HS has an overlapping presentation with cytokine release syndrome (CRS) and immune effector cell-associated neurotoxicity syndrome (ICANS) but the American Society for Transplantation and Cellular Therapy (ASTCT) defines it as a distinct entity [77,78]. IEC-HS is driven by IFN-γ and preliminary data suggest that CXCL9 may help identify patients at risk and guide early intervention [79]. As CAR T-cell therapies expand across hematologic malignancies, understanding how CXCL9 behaves in this context—and whether existing HLH-derived thresholds apply—will provide highly useful insight.

Finally, future work should clarify whether CXCL9 can guide treatment decisions, particularly targeted treatment selection. The emerging distinction between CXCL9 as a marker of disease severity and CXCL9 as a marker of IFN-γ-targetable biology is clinically important. Trials and well-designed registries should test whether CXCL9-enriched populations derive greater benefit from IFN-γ-directed therapy, JAK inhibition, or combination approaches, and whether early CXCL9 normalization predicts durable remission. Such studies would help determine whether CXCL9 can evolve from a descriptive biomarker into a practical tool for individualized HLH management.

## 7. Conclusions

CXCL9 is a compelling biomarker for recognizing HLH and guiding treatment. The currently available evidence supports its use as an adjunctive marker for distinguishing HLH from selected mimics, identifying patients at higher risk of early mortality, monitoring response to IFN-γ-directed therapy, and detecting possible reactivation. Its value is strongest when interpreted as part of a broader clinical and laboratory assessment rather than in isolation or as a replacement for established diagnostic criteria. However, there remains important limitations, mostly surrounding the lack of multicenter prospective and validated studies. Limitations such as the lack of universal cutoff values and poor cross-assay standardization reinforce the need for careful interpretation by clinical context, HLH subtype, age, alternative diagnoses, and timing relative to treatment. Nevertheless, CXCL9 offers an important step forward within HLH diagnostics. By linking a measurable serum marker to a therapeutically targetable immune pathway, CXCL9 may help clinicians move beyond nonspecific markers of inflammation toward biologically informed diagnosis, prognostication, and treatment monitoring.

## Figures and Tables

**Table 1:** Diagnostic frameworks for familial (primary) and secondary HLH.

Characteristic	HLH-2004 [11]	HLH-2024 [12]	HScore (Points) ^1^ [10]
Population studied in	Pediatric (familial HLH)	Pediatric (familial HLH)	Adult (secondary HLH)
Diagnostic threshold	≥5 of 8 ^2^	≥5 of 7 ^2^	≥168 points
Underlying immunosuppression	-	-	Included (+18)
Fever	>38.5 °C	≥38.5 °C	<38.4 °C (0) 38.4–39.4 °C (+33) >39.4 °C (+49)
Splenomegaly	Present (no size cutoff)	≥2 cm below the costal margin	None (0) One (+23) Both (+38)
Hepatomegaly	-	-	
Cytopenias	≥2 of 3 lineages ^3^	≥2 of 3 lineages ^4^	1 lineage (0) 2 lineages (+24) 3 lineages ^5^ (+34)
Triglycerides	Fasting triglycerides ≥ 265 mg/dL	Fasting triglycerides ≥ 265 mg/dL	<132.7 mg/dL (0) 132.7–354 mg/dL (+44) >354 mg/dL (+64)
Fibrinogen	≤150 mg/dL	≤150 mg/dL	>250 mg/dL (0) ≤250 mg/dL (+30)
NK cell activity	Low or absent	-	-
Ferritin	≥500 µg/L	≥500 µg/L	<2000 ng/mL (0) 2000–6000 ng/mL (+35) >6000 ng/mL (+50)
sCD25	≥2400 U/mL	≥2400 U/mL	-
Aspartate aminotransferase	-	-	<30 U/L (0) ≥30 U/L (+19)
Hemophagocytosis	Present in bone marrow, spleen, or lymph nodes without evidence of malignancy	Present	Present on bone marrow aspirate (+35)
Molecular diagnosis	Separate pathway ^5^	Separate pathway ^5^	-
Cytotoxicity assay	-	Separate pathway ^5^	-
CXCL9	-	-	-

^1^ The best cutoff value for HScore was 169, corresponding to a sensitivity of 93%, specificity of 86%, and accurate classification of 90% of patients. ^2^ Or molecular diagnosis, genetic confirmation, or cytotoxicity assay confirmation. ^3^ Hemoglobin < 9 g/dL, platelet < 100,000/µL, absolute neutrophil count < 1.0 × 10^9^/L. ^4^ Hemoglobin ≤ 9.2 g/dL, platelet ≤ 110,000/µL, white blood cell ≤ 5000/µL. ^5^ HLH-2004 and HLH-2024 report several separate diagnostic pathways for HLH using molecular/genetic testing as well as various cytotoxicity markers.

**Table 2:** Summary of proposed CXCL9 cutoff values for the diagnosis and prognosis of HLH.

Study	Population	*n* (HLH+)	Proposed Cutoff	Intended Use	Context
Maruoka et al., 2014 [44]	Adults with B-cell (60%) and T/NK-cell (40%) lymphoma-associated HLH.	15	>5000 pg/mL	Diagnostic	Sensitivity 100% and specificity 95% for the diagnosis of lymphoma-associated HLH.
Debaugnies et al., 2021 [33]	Adults with infection (43%), infection + malignancy (21%), malignancy (29%), and unknown etiology (7%) HLH	14	>514 pg/mL	Diagnostic	Optimal cutoff by Youden index: sensitivity 64% and specificity 91% in HLH+ patients. AUC was 0.7540 for discriminating HLH from mimicking diagnoses.
Johnson et al., 2025 [45]	Hospitalized adults with HLH (malignancy 52%, infection 12%, transplant 8%, rheumatologic 5%, and other 23%) treated with emapalumab or ruxolitinib.	77	>3500 pg/mL	Prognostic	Pre-treatment CXCL9 level predictive of improved overall survival in HLH patients receiving emapalumab and/or ruxolitinib.
Lan et al., 2025 [46]	Adult and pediatric patients with refractory HLH (EBV 60%, autoimmune disease 13%, non-EBV infection 7%, familial 7%, and other) who received RED therapy.	15	No discrete cutoff	Prognostic	Out of 11 cytokines tested, only baseline CXCL9 and IL-18 levels were significantly higher in patients who went on to respond to RED therapy.
Luo et al., 2025 [18]	Pediatric patients with EBV (72%), familial (8%), malignancy (4%), MAS (4%), and other HLH etiologies.	53	Elevated	Diagnostic	Elevated CXCL9 had a sensitivity of 98.1% in HLH+ patients.
Rocco et al., 2026 [32]	Hospitalized patients ≥ 15 years old with HLH (malignancy 24%, infection + malignancy 18%, rheumatologic 11%, infection 14%, CAR-T 9%, HCT 4%, IEI 5%, other 15%).	126	>16,100 pg/mL	Prognostic	CXCL9 > 16,100 pg/mL was significantly associated with 90-day mortality and continuously rising CXCL9 was also associated with higher mortality.

**Table 3:** Key biomarkers representing the biological pathways that drive HLH.

Biomarker	Pathway	Strengths	Limitations	References
Ferritin	Macrophage activation and iron storage	Highly sensitive; included in HLH-2004, HLH-2024, and the HScore	Relatively poor specificity; elevated in iron overload, malignancy, renal disease, sepsis, and more	[3,21]
sCD25	NK and T-cell activation	High sensitivity; sCD25:ferritin ratio helpful in lymphoma-associated HLH; included in HLH-2004 and HLH-2024	Imperfect specificity; elevated in lymphoma, HIV, other immune activation disorders	[3,7,21,36]
IL-18	Inflammasome activation	Helpful in distinguishing autoinflammatory-related HLH	Not included in any validated frameworks	[7,14,21,36,46]
CXCL9	IFN-γ	Near-universal positivity in HLH; unique to IFN-γ-driven disease; can help guide treatment	Lacks laboratory standardization; not included in any validated frameworks	[18,19,21,31,32,36,44,45,46]

## Data Availability

No new data were created or analyzed in this study. Data sharing is not applicable to this article.

## Abbreviations

The following abbreviations are used in this manuscript:

HLH	Hemophagocytic lymphohistiocytosis
CXCL	C-X-C motif chemokine ligand
NK	Natural killer
MAS	Macrophage activation syndrome
CAR	Chimeric antigen receptor
IFN	Interferon
CXCR	C-X-C motif chemokine receptor
AST	Aspartate aminotransferase
EULAR	European Alliance of Associations for Rheumatology
ACR	American College of Rheumatology
EBV	Epstein–Barr virus
IQR	Interquartile range
pg	Picogram
mL	Milliliter
CNS	Central nervous system
CSF	Cerebrospinal fluid
ONID	Other neuroinflammatory disorders
ng	Nanogram
AUC	Area under the curve
SEBVTCL	Systemic Epstein–Barr virus-positive T-cell lymphoma of childhood
CAEBV	Chronic active Epstein–Barr virus disease
TCR	T-cell receptor
WHO	World Health Organization
NACHO	North American Consortium for Histiocytosis
HR	Hazard ratio
CI	Confidence interval
RED	Ruxolitinib, emapalumab, dexamethasone
CRP	C-reactive protein
ELISA	Enzyme-linked immunosorbent assay
FDA	Food and Drug Administration
GVHD	Graft-versus-host disease
IEC-HS	Immune effector cell-associated HLH-like syndrome
CRS	Cytokine release syndrome
ICANS	Immune effector cell-associated neurotoxicity syndrome
ASTCT	American Society for Transplantation and Cellular Therapy

## References

[1] Scott R.B., Robb-Smith A.H.T. (1939). Histiocytic Medullary Reticulosis. Lancet.

[2] Farquhar J.W., Claireaux A.E. (1952). Familial Haemophagocytic Reticulosis. Arch. Dis. Child..

[3] Jordan M.B., Allen C.E., Weitzman S., Filipovich A.H., Mcclain K.L. (2011). How I treat hemophagocytic lymphohistiocytosis. Blood.

[4] Kaushansky K., Prchal J.T., Burns L.J., Lichtman M.A., Levi M., Linch D.C. (2021). Williams Hematology.

[5] Hsu J.I., Nikiforow S., Berliner N. (2026). Hemophagocytic lymphohistiocytosis in adults. Blood.

[6] Tan C.J., Ng Z.Q., Bhattacharyya R., Sultana R., Lee J.H. (2023). Treatment and mortality of hemophagocytic lymphohistiocytosis in critically ill children: A systematic review and meta-analysis. Pediatr. Blood Cancer.

[7] Henter J.-I. (2025). Hemophagocytic Lymphohistiocytosis. N. Engl. J. Med..

[8] Lorenzen M.O.B., Jensen M., Jørgensen R.R.K., Jensen P., Frederiksen H., Hansen D.L., El Fassi D., Andersen M.N., El-Galaly T.C. (2026). Incidence and Survival of Hemophagocytic Lymphohistiocytosis Over Two Decades: A Population-Based Study. Eur. J. Haematol..

[9] Wang C.-Y., Lee J.-H., Lee N.-C., Hu Y.-C., Chang H.-H., Wang L.-C., Lin Y.-T., Yang Y.-H., Chiang B.-L., Yu H.-H. (2025). Etiologies and long-term outcome of pediatric hemophagocytic lymphohistiocytosis and macrophage activation syndrome in Taiwan: A single-center retrospective study. Front. Immunol..

[10] Fardet L., Galicier L., Lambotte O., Marzac C., Aumont C., Chahwan D., Coppo P., Hejblum G. (2014). Development and validation of the HScore, a score for the diagnosis of reactive hemophagocytic syndrome. Arthritis Rheumatol..

[11] Henter J., Horne A., Arico M., Egeler R., Filipovich A., Imashuku S., Ladisch S., McClain K., Webb D., Winiarski J. (2007). HLH-2004: Diagnostic and therapeutic guidelines for hemophagocytic lymphohistiocytosis. Pediatr. Blood Cancer.

[12] Henter J.-I., Sieni E., Eriksson J., Bergsten E., Hed Myrberg I., Canna S.W., Coniglio M.L., Cron R.Q., Kernan K.F., Kumar A.R. (2024). Diagnostic guidelines for familial hemophagocytic lymphohistiocytosis revisited. Blood.

[13] Ramos-Casals M., Brito-Zerón P., López-Guillermo A., Khamashta M.A., Bosch X. (2014). Adult haemophagocytic syndrome. Lancet.

[14] Chiossone L., Audonnet S., Chetaille B., Chasson L., Farnarier C., Berda-Haddad Y., Jordan S., Koszinowski U.H., Dalod M., Mazodier K. (2012). Protection from Inflammatory Organ Damage in a Murine Model of Hemophagocytic Lymphohistiocytosis Using Treatment with IL-18 Binding Protein. Front. Immunol..

[15] Tokunaga R., Zhang W., Naseem M., Puccini A., Berger M.D., Soni S., McSkane M., Baba H., Lenz H.-J. (2018). CXCL9, CXCL10, CXCL11/CXCR3 axis for immune activation—A target for novel cancer therapy. Cancer Treat. Rev..

[16] Lee A.J., Ashkar A.A. (2018). The Dual Nature of Type I and Type II Interferons. Front. Immunol..

[17] Groom J., Luster A. (2011). CXCR3 ligands: Redundant, collaborative and antagonistic functions. Immunol. Cell Biol..

[18] Luo Z., Chen Y., Gu M., Zhao F., Liang J., Tang Y., Xu X. (2025). Interferon-gamma and C-X-C motif chemokine ligand 9, which is better for haemophagocytic lymphohistiocytosis diagnosis and monitoring?. Br. J. Haematol..

[19] Prencipe G., Bracaglia C., Caiello I., Pascarella A., Francalanci P., Pardeo M., Meneghel A., Martini G., Rossi M.N., Insalaco A. (2019). The interferon-gamma pathway is selectively up-regulated in the liver of patients with secondary hemophagocytic lymphohistiocytosis. PLoS ONE.

[20] Diamond T., Lau M., Morrissette J., Chu N., Behrens E.M. (2023). CXCL9 inhibition does not ameliorate disease in murine models of both primary and secondary hemophagocytic lymphohistiocytosis. Sci. Rep..

[21] Shakoory B., Geerlinks A., Wilejto M., Kernan K., Hines M., Romano M., Piskin D., Ravelli A., Sinha R., Aletaha D. (2023). The 2022 EULAR/ACR Points to Consider at the Early Stages of Diagnosis and Management of Suspected Haemophagocytic Lymphohistiocytosis/Macrophage Activation Syndrome (HLH/MAS). Arthritis Rheumatol..

[22] Bracaglia C., de Graaf K., Pires Marafon D., Guilhot F., Ferlin W., Prencipe G., Caiello I., Davì S., Schulert G., Ravelli A. (2017). Elevated circulating levels of interferon-γ and interferon-γ-induced chemokines characterise patients with macrophage activation syndrome complicating systemic juvenile idiopathic arthritis. Ann. Rheum. Dis..

[23] Mizuta M., Shimizu M., Inoue N., Nakagishi Y., Yachie A. (2019). Clinical significance of serum CXCL9 levels as a biomarker for systemic juvenile idiopathic arthritis associated macrophage activation syndrome. Cytokine.

[24] Han J.H., Suh C.H., Jung J.Y., Ahn M.H., Han M.H., Kwon J.E., Yim H., Kim H.A. (2017). Elevated circulating levels of the interferon-γ-induced chemokines are associated with disease activity and cutaneous manifestations in adult-onset Still’s disease. Sci. Rep..

[25] Arger N.K., Ho M.E., Allen I.E., Benn B.S., Woodruff P.G., Koth L.L. (2020). CXCL9 and CXCL10 are differentially associated with systemic organ involvement and pulmonary disease severity in sarcoidosis. Respir. Med..

[26] Sampath P., Rajamanickam A., Thiruvengadam K., Natarajan A.P., Hissar S., Dhanapal M., Thangavelu B., Jayabal L., Ramesh P.M., Ranganathan U.D. (2023). Plasma chemokines CXCL10 and CXCL9 as potential diagnostic markers of drug-sensitive and drug-resistant tuberculosis. Sci. Rep..

[27] Setiadi A., Zoref-Lorenz A., Lee C.Y., Jordan M.B., Chen L.Y.C. (2022). Malignancy-associated haemophagocytic lymphohistiocytosis. Lancet Haematol..

[28] De Benedetti F., Prencipe G., Bracaglia C., Marasco E., Grom A.A. (2021). Targeting interferon-γ in hyperinflammation: Opportunities and challenges. Nat. Rev. Rheumatol..

[29] Nikčević A., Matotek L.R., Šiftar S., Marić L.S., Tešović G., Zidovec-Lepej S., Nikčević A., Radmanić Matotek L., Šiftar S., Stemberger Marić L. (2026). Increased Expression of CXCL9, CXCL10, and CXCL11 in Epstein–Barr Virus-Associated Infectious Mononucleosis and the Role of CXCL5 as a Candidate Biomarker of Disease Severity. Pathogens.

[30] Yin X., Wang Z., Wu T., Ma M., Zhang Z., Chu Z., Hu Q., Ding H., Han X., Xu J. (2019). The combination of CXCL9, CXCL10 and CXCL11 levels during primary HIV infection predicts HIV disease progression. J. Transl. Med..

[31] Kaneko S., Hatano M., Shimbo A., Miyaoka F., Irabu H., Akutsu Y., Hayashi Y., Mizuta M., Nakagishi Y., Akamine K. (2026). Serum Cytokine Profiling Differentiates Underlying Diseases in Cytokine Storm Syndrome. Arthritis Rheumatol..

[32] Rocco J., Oved J., Patel R., Herskovits A., Nair N., Shakoory B., Robertson T., Dileo R., Jacobs M., Zerbe C. (2026). CXCL9 as a novel prognostic marker to identify high-risk adults with hemophagocytic lymphohistiocytosis. Blood.

[33] Debaugnies F., Mahadeb B., Nagant C., Meuleman N., De Bels D., Wolff F., Gottignies P., Salaroli A., Borde P., Voué M. (2021). Biomarkers for Early Diagnosis of Hemophagocytic Lymphohistiocytosis in Critically Ill Patients. J. Clin. Immunol..

[34] Kim M.-M., Yum M.-S., Choi H.-W., Ko T.-S., Im H.J., Seo J.-J., Koh K.-N. (2012). Central nervous system (CNS) involvement is a critical prognostic factor for hemophagocytic lymphohistiocytosis. Korean J. Hematol..

[35] Chandra S., Greis K.D., Chutipongtanate S., Luebbering N., Webster A., Poisson K.E., Fisher K.S., Smiley K., Marsh R.A., Jordan M.B. (2026). Novel CSF biomarkers for facilitating diagnosis of CNS-HLH from other neuroinflammatory disorders. Blood Adv..

[36] Zhao Y., Ou W., Wei A., Ma H., Zhang L., Lian H., Zhang Q., Wang D., Li Z., Zhang R. (2024). Biomarkers in Pediatric Hemophagocytic Lymphohistiocytosis with Central Nervous System Involvement: A Cohort Study. J. Pediatr. Hematol./Oncol..

[37] Lin H., Scull B.P., Goldberg B.R., Abhyankar H.A., Eckstein O.E., Zinn D.J., Lubega J., Agrusa J., El Mallawaney N., Gulati N. (2021). IFN-γ signature in the plasma proteome distinguishes pediatric hemophagocytic lymphohistiocytosis from sepsis and SIRS. Blood Adv..

[38] Alaggio R., Amador C., Anagnostopoulos I., Attygalle A.D., Araujo I.B.O., Berti E., Bhagat G., Borges A.M., Boyer D., Calaminici M. (2022). The 5th edition of the World Health Organization Classification of Haematolymphoid Tumours: Lymphoid Neoplasms. Leukemia.

[39] Coffey A.M., Lewis A., Marcogliese A.N., Elghetany M.T., Punia J.N., Chang C.-C., Allen C.E., McClain K.L., Gaikwad A.S., El-Mallawany N.K. (2019). A clinicopathologic study of the spectrum of systemic forms of EBV-associated T-cell lymphoproliferative disorders of childhood: A single tertiary care pediatric institution experience in North America. Pediatr. Blood Cancer.

[40] Sato Y., Okuno Y., Murata T., Kimura H. (2026). Epstein–Barr Virus-Associated T/NK-Cell Neoplasms. J. Med. Virol..

[41] Matsuda K., Nakazawa Y., Yanagisawa R., Honda T., Ishii E., Koike K. (2011). Detection of T-cell receptor gene rearrangement in children with Epstein-Barr virus-associated hemophagocytic lymphohistiocytosis using the BIOMED-2 multiplex polymerase chain reaction combined with GeneScan analysis. Clin. Chim. Acta.

[42] Jordan M.B. (2024). Hemophagocytic lymphohistiocytosis: A disorder of T cell activation, immune regulation, and distinctive immunopathology. Immunol. Rev..

[43] Jordan M., Allen C., Greenberg J., Henry M., Hermiston M., Kumar A., Hines M., Eckstein O., Ladisch S., Nichols K. (2019). Challenges in the diagnosis of hemophagocytic lymphohistiocytosis: Recommendations from the North American Consortium for Histiocytosis (NACHO). Pediatr. Blood Cancer.

[44] Maruoka H., Inoue D., Takiuchi Y., Nagano S., Arima H., Tabata S., Matsushita A., Ishikawa T., Oita T., Takahashi T. (2014). IP-10/CXCL10 and MIG/CXCL9 as novel markers for the diagnosis of lymphoma-associated hemophagocytic syndrome. Ann. Hematol..

[45] Johnson W., Rocco J., Patel R., Herskovits A., Mead E., Park J., Moskowitz A., Epstein-Peterson Z., Stuver R., Bapodra N. (2025). CXCL9 is a predictive biomarker of survival with IFNγ-targeted therapy in adult HLH patients. Blood.

[46] Lan X., Wei N., Wang J., Wang Z. (2025). CXCL9 and IL-18: Potential biomarkers for efficacy evaluation in refractory hemophagocytic lymphohistiocytosis treated with RED (ruxolitinib, emapalumab and dexamethasone). Ann. Hematol..

[47] Landy E., Carol H., Ring A., Canna S. (2023). Biological and clinical roles of IL-18 in inflammatory diseases. Nat. Rev. Rheumatol..

[48] Wojtovicova T., Aujesky D., Schefold J.C., Daskalakis M., Furrer H., Novak U., Pabst T., Maurer B., Möller B., Abegglen R.C. (2025). Hemophagocytic Syndromes in Adults: Real-World Data on Mortality from a Tertiary Reference Center. Acta Haematol..

[49] La Marle S., Richard-Colmant G., Fauvernier M., Ghesquières H., Hot A., Sève P., Jamilloux Y. (2023). Mortality and Associated Causes in Hemophagocytic Lymphohistiocytosis: A Multiple-Cause-of-Death Analysis in France. J. Clin. Med..

[50] Locatelli F., Jordan M.B., Allen C., Cesaro S., Rizzari C., Rao A., Degar B., Garrington T.P., Sevilla J., Putti M.-C. (2020). Emapalumab in Children with Primary Hemophagocytic Lymphohistiocytosis. N. Engl. J. Med..

[51] Zoref-Lorenz A., Allen C.E., Behrens E.M., Chandrakasan S., Chien M., Hermiston M.L., Hinson A.P., Intzes S., Isakoff M.S., Leiding J.W. (2026). Emapalumab use in malignancy-associated hemophagocytic lymphohistiocytosis in the United States: The REAL-HLH study. Blood Adv..

[52] Rodrigues J.B., Nasr B.P., Cypriano M.d.S. (2021). Hemophagocytic lymphohistiocytosis: Presentation and outcome of twenty-one patients at a single institution. Hematol. Transfus. Cell Ther..

[53] La Rosée P., Horne A., Hines M., von Bahr Greenwood T., Machowicz R., Berliner N., Birndt S., Gil-Herrera J., Girschikofsky M., Jordan M.B. (2019). Recommendations for the management of hemophagocytic lymphohistiocytosis in adults. Blood.

[54] Herskovits A.Z., Johnson W.T., Oved J.H., Irwin S., Doddi S., John D., Ocasio A., Ramanathan L.V. (2024). A semiautomated microfluidic ELISA for the detection of hemophagocytic lymphohistiocytosis biomarkers. Am. J. Clin. Pathol..

[55] Systems R.D. Human CXCL9/MIG Quantikine ELISA Kit Catalog #DCX900, Minneapolis, MN. https://www.rndsystems.com/target/cxcl9-mig/elisa-kits?brand_facet=rnd&gclsrc=aw.ds&gad_source=1&gad_campaignid=16960280051&gbraid=0AAAAAD_kmX3creMQIaH9Dim96xd8tPFr2&gclid=CjwKCAjw48TUBhBREiwAK0GnQZkXqhERdQ0tlWPtHdOU0BgC1FCAWaBrkLj_tKWfs-sRYYbM9KjbxRoC7FcQAvD_BwE.

[56] Systems R.D. Human Luminex^®^ Discovery Assay Catalog #LXSAHM. https://www.rndsystems.com/products/human-luminex-discovery-assay_lxsahm.

[57] ProteinSimple Ella Automated Immunoassay System Catalog #600-100. https://www.medikonia.com/product_view.php?id=1561.

[58] Hasegawa T., Yoshida M., Watanabe S., Kondo T., Asada H., Nakagawa A., Tomii K., Kameda M., Otsuka M., Kuronuma K. (2023). Development of a new HISCL automated CXCL9 immunoassay. Sci. Rep..

[59] Franco-Dilone L., Wong O.A., Laterza O.F., Zhao X. (2025). A clinical biomarker assay to quantitate CXCL9 in human serum. BioTechniques.

[60] Sysmex Automated Immunoassay System HISCL 800/5000. https://www.sysmex-europe.com/products/products-detail/hiscl/.

[61] Rountree W., Lynch H.E., Denny T.N., Sempowski G.D., Macintyre A.N. (2024). Sources of variability in Luminex bead-based cytokine assays: Evidence from twelve years of multi-site proficiency testing. J. Immunol. Methods.

[62] van Meijgaarden K., Khatri B., Smith S., Drittij A., de Paus R., Goeman J., Ho M., Dockrell H., McShane H., Joosten S. (2018). Cross-laboratory evaluation of multiplex bead assays including independent common reference standards for immunological monitoring of observational and interventional human studies. PLoS ONE.

[63] Knight V., Long T., Meng Q., Linden M., Rhoads D. (2020). Variability in the Laboratory Measurement of Cytokines. Arch. Pathol. Lab. Med..

[64] Ionescu M., Prajescu B., Taras R., Popescu N., Vidlescu R., Smarandoiu M., Rosca L.-E., Enculescu A., Berghea E., Toma C. (2024). Diagnostic Challenges in Hemophagocytic Lymphohistiocytosis, a Rare, Potentially Fatal Disease: Two Case Studies. J. Clin. Med..

[65] Otrock Z., Daver N., Kantarjian H., Eby C. (2017). Diagnostic Challenges of Hemophagocytic Lymphohistiocytosis. Clin. Lymphoma Myeloma Leuk..

[66] Giesen N., Schwarzbich M., Dischinger K., Becker N., Hummel M., Benner A., Radujkovic A., Müller-Tidow C., Dreger P., Luft T. (2020). CXCL9 Predicts Severity at the Onset of Chronic Graft-versus-host Disease. Transplantation.

[67] Kitko C.L., Levine J.E., Storer B.E., Chai X., Fox D.A., Braun T.M., Couriel D.R., Martin P.J., Flowers M.E., Hansen J.A. (2014). Plasma CXCL9 elevations correlate with chronic GVHD diagnosis. Blood.

[68] Srikantan S., Ma X., Alavi A., Emole J., Peres E., Abidi M., Farhan S. (2026). CXCL9, an Early Inflammatory Marker As Prognostic Indicator Following Allogeneic Stem Cell Transplantation: A Single Prospective cohort study. Transplant. Cell. Ther..

[69] Bolomsky A., Schreder M., Zojer N., Ludwig H. (2015). The chemokine CXCL9 (MIG) is an independent predictor of overall survival in newly diagnosed multiple myeloma. Clin. Lymphoma Myeloma Leuk..

[70] Giamarellos-Bourboulis E., Antonelli M., Bloos F., Kotsamidi I., Psarrakis C., Dakou K., Thomas-Rüddel D., Montini L., Briegel J., Damoraki G. (2024). Interferon-gamma driven elevation of CXCL9: A new sepsis endotype independently associated with mortality. EBioMedicine.

[71] Ravalet N., Guermouche H., Hirsch P., Picou F., Foucault A., Gallay N., Martignoles J., Beaud J., Suner L., Deswarte C. (2023). Modulation of bone marrow and peripheral blood cytokine levels by age and clonal hematopoiesis in healthy individuals. Clin. Immunol..

[72] Brito-de-Sousa J., Lima-Silva M., Costa-Rocha I., Campi-Azevedo A., Mambrini J., Faria A., Lima-Costa M., Peixoto S., Teixeira-Carvalho A., Torres K. (2025). Rhythms and shifts of chemokines and cytokines interplay in a decade lifespan: The longitudinal community-based Bambuí health and aging study. Exp. Gerontol..

[73] Torres K., Rezende V., Lima-Silva M., Santos L., Costa C., Mambrini J., Peixoto S., Tarazona-Santos E., Martins Filho O., Lima-Costa M. (2018). Immune senescence and biomarkers profile of Bambuí aged population-based cohort. Exp. Gerontol..

[74] Larsson A., Carlsson L., Gordh T., Lind A., Thulin M., Kamali-Moghaddam M. (2015). The effects of age and gender on plasma levels of 63 cytokines. J. Immunol. Methods.

[75] Sepiashvili L., Alli Z., Bohn M.K., Hall A., Karin A., Murata K., Adeli K. (2021). Complex biological patterns of soluble cytokines and CD163 in childhood necessitating age-specific reference intervals for evidence-based clinical interpretation. Clin. Biochem..

[76] Ombada M., Sandhu M. (2024). Immune Effector Cell-Associated HLH-Like Syndrome as a Post CAR T-Cell Therapy Complication of Lymphoid Malignancies. American Society of Hematology Morning Report.

[77] Hines M., Knight T., McNerney K., Leick M., Jain T., Ahmed S., Frigault M., Hill J., Jain M., Johnson W. (2023). Immune Effector Cell-Associated Hemophagocytic Lymphohistiocytosis-Like Syndrome. Transplant. Cell. Ther..

[78] Sterner R.C., Sterner R.M. (2022). Immune effector cell associated neurotoxicity syndrome in chimeric antigen receptor-T cell therapy. Front. Immunol..

[79] Srinagesh H., Kramer A., Baird J., Reschke A., Sahaf B., Cancilla J., Syal S., Su Y., Agarwal N., Jensen A. (2026). Clinical and Cytokine Features of Immune Effector Cell-Associated Hemophagocytic Lymphohistiocytosis-Like Syndrome. Blood Cancer Discov..

